# Influence of Tool Geometry and Process Parameters on Torque, Temperature, and Quality of Friction Stir Welds in Dissimilar Al Alloys

**DOI:** 10.3390/ma14206020

**Published:** 2021-10-13

**Authors:** Neves Manuel, Daniel Beltrão, Ivan Galvão, Rui M. Leal, José D. Costa, Altino Loureiro

**Affiliations:** 1 Univ Coimbra, CEMMPRE, Departamento de Engenharia Mecânica, Rua Luís Reis Santos, 3030-788 Coimbra, Portugal; uc2013112368@student.uc.pt (N.M.); uc201966996@student.uc.pt (D.B.); rui.leal@dem.uc.pt (R.M.L.); jose.domingos@dem.uc.pt (J.D.C.); altino.loureiro@dem.uc.pt (A.L.); 2 Faculdade de Engenharia e Tecnologia, Universidade do Namibe, Campus Farol Noronha, Moçâmedes 274, Angola; 3 ISEL, Departamento de Engenharia Mecânica, Instituto Politécnico de Lisboa, Rua Conselheiro Emídio Navarro 1, 1959-007 Lisboa, Portugal; 4 LIDA-ESAD.CR, Instituto Politécnico de Leiria, Rua Isidoro Inácio Alves de Carvalho, 2500-321 Caldas da Rainha, Portugal

**Keywords:** friction stir welding, tool geometry, dissimilar aluminium alloys, torque, temperature

## Abstract

In the current investigation, the influence of the tool geometry, the position of the materials in the joint, the welding speed on the temperature and torque developed, and on the quality of the welds in dissimilar and tri-dissimilar T joints were analysed. The aluminium alloys used were AA2017-T4, AA6082-T6, and AA5083-H111 and the friction stir welds were performed with identical shoulder tools, but with either a pin with simple geometry or a pin with progressive geometry. Progressive pin tools proved to be a viable alternative in the production of dissimilar and tri-dissimilar welds, as they provide a larger tool/material friction area and a larger volume of dragged material, which promotes an increase in the heat generated and a good mixing of the materials in the stir zone, although they require a higher torque. Placing a stronger material on the advancing side also results in a higher temperature in the stir zone but requires higher torque too. The combination of these factors showed that tools with a progressive pin provide sound dissimilar and tri-dissimilar welds, unlike single-pin tools. The increase in the welding speed causes the formation of defects in the stir zone, even in tri-dissimilar welds carried out with a tool with a progressive pin, which impairs the fatigue strength of the welds.

## 1. Introduction

There is a trend in shipbuilding of increasingly using aluminium alloys in boat building to reduce weight, increase payload, and reduce the fuel consumption of ships. Aluminium alloys of the 5xxx and 2xxx series are used in the submerged hulls of the ships, while the superstructures are typically produced in 6xxx alloys. To increase the stiffness of the naval structures, without significantly increasing the weight, the plates are usually reinforced with a stringer, giving rise to T-joints. However, the production of aluminium T-joints by conventional fusion-welding techniques may lead to weaknesses, often promoting significant distortion and the formation of metallurgical defects in the welded components [1]. However, friction stir welding (FSW) is a very suitable alternative to produce T-joints in aluminium alloys. As it is a solid-state technology, the heat-input during the process is much lower than in conventional techniques, which results in a significant reduction in the metallurgical problems occurring during welding and enables the production of components with good dimensional stability and lower distortion [2].

The characteristics of the FS welds are determined by the thermomechanical conditions experienced by the materials during welding. These conditions, which are closely related to the generation of strain and heat, significantly influence the material flow and the weld microstructure [3]. As a result, it is mandatory to understand the influence of the welding parameters, such as: the tool rotation and welding speeds, the tool geometry, and the base material properties on the thermomechanical conditions. The optimisation of the welding parameters has been extensively studied with the analysis of microstructural and mechanical properties in similar and dissimilar FSW [4,5]. Welding torque and temperature have also been used as parameters to control the welding results due to their strong relation to the heat generated during the process [6,7]. Yan et al. [8] reported that the torque increases in increments with the welding speed and low torque values are associated with high heat-input conditions. In good agreement with this, Arora et al. [9] explained that the decrease in torque for lower welding speeds is associated with the decrease in the volume of material being deformed by the tool with each revolution. As the heat is generated in a smaller volume, this leads to a higher temperature in the material and a lower flow stress. According to Leitão et al. [10], the main factors governing the torque values are the rotation speed and the plate’s thickness because of their influence on the generation of heat and the distribution of heat throughout the thickness and flow of materials. These authors also reported that the plastic properties of the base materials also have a strong impact on the torque values. 

The influence of the tool geometry on the welding torque has also been addressed in the literature. For example, Mehta et al. [11] investigated the torque and temperature in friction stir welding to optimise the diameter of the tool shoulder. In turn, Papahn et al. [12] compared the evolution of torque for three different tool geometries (triangular, non-threaded cylindrical, and threaded tapered pin). The authors observed that a lower torque was registered when a threaded tapered pin was used to produce the welds. Additionally, they observed that the diameter of the tool shoulder was directly correlated with the temperature reached, axial force, and the weld’s characteristics.

Recently, Andrade et al. [13], based on numerical simulation and published experimental results, concluded that the tool’s dimensions and its rotational speed directly influence the torque and temperature reached during the welding. Torque decreases with increasing tool rotation speed, while temperature increases with the tool rotation speed and the tool friction area. They also concluded that the welding speed and thickness of the base material are secondary parameters of torque and temperature. Most of the studies mentioned above refer to butt welds in the same material, therefore similar welds. 

Regarding dissimilar welds, most studies focus on the influence of the welding parameters and the relative position of materials (advancing or retreating side) on the morphology and mechanical strength of welds and only a few on torque but, in any case, results are scarce and not always in agreement. Barbini et al. [14] stated, concerning the butt welding of AA7050-T7651 and AA2024-T3 alloys, that welds with a better quality and a greater mechanical resistance are obtained when the less resistant alloy (AA2024-T3) is placed on the advancing side. Additionally, note that when the AA7050-T7651 alloy is placed on the advancing side, there is a general reduction in torque. On the other hand, Cavalieire et al. [15] reported that higher torques and forces are registered when the strongest alloy, AA2024, is placed on the advancing side when welded to AA6082. However, Elnabi et al. [16] reported, using statistical analysis, that in dissimilar welding of AA5454 to AA7075, the location of the base metal has negligible importance regarding the strength of the welds.

Concerning the production of T-joints by FSW, few studies have been conducted. Jesus et al. [17] showed the influence of the tool geometry on the morphology and mechanical behaviour of similar welds in T-joint configuration in the AA6082 and AA5083 alloys. Astarita et al. [18], analysing the feasibility of dissimilar T joints of AA 6056-T4 and AA 2198-T3 by FSW, discovered flow defects, such as the lack of metallurgical continuity between skin and stringer, which were found to play a significant role in both mechanical and electrochemical behaviour. Tavares et al. [19] investigated the feasibility and mechanical behaviour of welds with this geometry, between the AA7075-T6 (stringer) and AA6056-T4 (skin) alloys. They found that the welds had good mechanical strength and a slight reduction in fatigue behaviour. More recently, Manuel et al. [20] studied the nugget formation and the mechanical behaviour of tri-dissimilar welds in AA2017-T4, AA5083-H111, and AA6082-T6 aluminium alloys. They showed that a high rotation to traverse speed ratio improves the overall weld quality, while increments in welding speed influence the weld morphology and fatigue strength. However, no studies were found on the effect of the process parameters on the torque or temperature induced for this joint geometry, whether for similar or dissimilar welds.

Despite the research already performed concerning the FSW of dissimilar T-joints, studies focused on analysing the influence of the tool geometry on welding temperature and torque, and therefore on the quality and mechanical behaviour of the welds, are still very scarce for this type of joint. Considering the very close relationship between the process outputs and the thermomechanical conditions experienced during welding, the systematic production of dissimilar T-joints by FSW requires a profound study of the welding temperature and torque. Therefore, the aim of the current study was to correlate the torque and temperature registered during welding with the position of the base material, the process parameters, and tool geometry and their consequences with regard to the quality of the weld.

## 2. Materials and Methods

**Figure 1:**
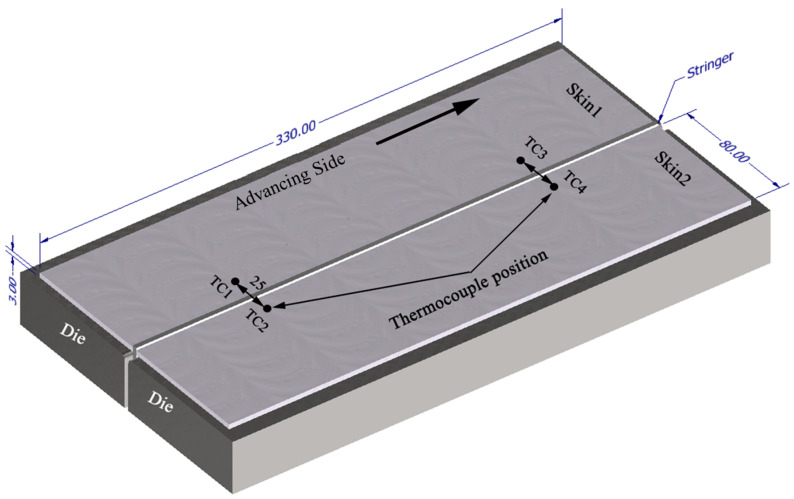
Welding setup and thermocouple location for the FSW joint.

T-joints in three different aluminium alloys, i.e., the heat-treatable AA2017-T4 and AA6082-T6 and the non-heat-treatable AA5083-H111, were produced by FSW, using ESAB Legio FSW 3UL equipment. The chemical composition and the mechanical properties of these alloys, which were obtained experimentally, are presented in Table 1 and Table 2, respectively. The joints were produced in the T-joint configuration, with the geometric characteristics illustrated in Figure 1. It may be observed that the joint is made of a skin, composed of two plates of 330 × 80 × 3 mm, and a stringer of 330 × 37.4 × 3 mm. The stringer was made to protrude 1.4 mm above the skin to provide enough material to fill the empty volumes between plates and dies in the fillets, avoiding any reduction in the skin’s thickness.

Dissimilar and tri-dissimilar welds were produced. For the dissimilar welds, two welding configurations were tested. The AA5083 alloy was positioned as the skin and the AA6082 alloy was positioned as the stringer in some joints, while the reverse welding configuration was adopted for the other joints. On the other hand, the AA6082 alloy was always positioned as the stringer plate in the tri-dissimilar welds, due to its good ability to deform plastically without creating cavities [21]. Even so, two welding configurations were also tested for these joints, which were associated with the relative position of the skin plates. Some welds were produced with the AA2017 alloy located on the advancing side and with the AA5083 alloy located on the retreating side and other welds were produced with the reverse positioning of the skin alloys. 

**Figure 2:**
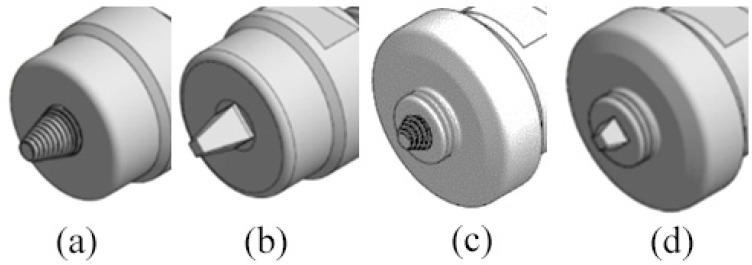
Tool designs tested: (**a**) TP—threaded pin; (**b**) PP—pyramidal pin; (**c**) PTP—progressive threaded pin; (**d**) PPP—progressive pyramidal pin.

As illustrated in Figure 2, four different tool designs were used to produce the welds. The tools, which were manufactured in quenched and tempered H13 steel, of 50HRC hardness, were composed of an 18 mm-diameter concave shoulder, the same for all tools, and a pin. Regarding the pin design, the tools were divided into two classes: with simple pin design and with progressive pin design. The simple pin tool had a threaded conical pin, designated (TP), and the other a flat pyramidal pin, designated (PP), both were 5.8 mm in length (Figure 2a,b). In turn, the progressive pin tools were composed of two different parts, i.e., a threaded cylindrical upper part that was 2.5 mm long plus a threaded conical tip (PTP) or a pyramidal tip (PPP), and both were 2.7 mm in length (Figure 2c,d).

As shown in Table 3, the variation in pin geometry changes the pin volume and the area of friction between the tool and the material. In this calculation, the area of the threads was not considered.

In the dissimilar welds, the influence of the tool geometry on morphology and its relationship with the torque required and the resulting temperature is mainly studied. For the tri-dissimilar welds, only the progressive threaded pin tool (PTP) was used. All welds were performed in position control in order to allow comparison with previous welds produced on a milling machine [21,22], where only the tool plunge depth was measured.

The welds were produced with a constant rotation speed (*w*) and tilt angle values of 500 rpm and 3°, respectively. Two traverse speeds (*v*) were tested for each tool and position of the materials, specifically, 60 and 120 mm/min for the dissimilar welds and 60 and 230 mm/min for the tri-dissimilar welds. *w*/*v* ratios of 8.3, 4.2, and 2.2 rot/mm correspond to welding speeds of 60, 120, and 230 mm/min, respectively. An increase in heat-input is usually associated with an increase in the *w*/*v* ratio [23]. These welding parameters, which were defined based on previous works [22], are shown in Table 4 and Table 5 for dissimilar and tri-dissimilar welds, respectively. The tool penetration depth was kept at 7 mm, with small variations in some series, to avoid the formation of surface defects. This penetration depth was measured relative to the top of the stringer. The nomenclature used to label the welds identifies the materials welded and their position in the joint, the welding tool, and the traverse speed. For example, the 56PP-60 label concerns a dissimilar weld produced with the AA5083 plate as the skin and the AA6082 plate as the stringer, using the progressive pyramidal tool and a traverse speed of 60 mm/min. In turn, 562TP-230 label corresponds to a tri-dissimilar weld produced with an AA6082 stringer, with the AA5083 and the AA2017 plates positioned on the advancing and retreating sides of the joint, respectively, using the progressive threaded pin tool and a traverse speed of 230 mm/min.

During welding, the torque was recorded by the welding equipment ESAB Legio FSW 3UL. Four K-type thermocouples were embedded in small holes distributed over the surface of the skin plates and close to the weld line to register the weld’s thermal cycles, as illustrated in Figure 1. A data translation device with an acquisition rate of 75 Hz and cold junction compensation was used to record the thermal cycles. After welding, all the welds were inspected visually. Then, the samples were removed transversely to the welding direction and prepared for metallographic analysis according to ASTM E3-11. Modified Keller’s and Weck’s reagents were used to etch the samples and a Leica DM4000M LED optical microscope was used to observe the morphology and the microstructure of the welds.

The microhardness of the welds was determined using an HMV-G SHIMADZU tester on the weld’s cross-section (along the skin and the stringer), with a testing load of 200 g for 15 s. Three lines of indentations were considered in each direction. The distance between the consecutive indentations was 1 mm. 

Three transverse tensile testing specimens were removed from each weld and machined according to the ASTM E8/E8M standard for testing metallic materials [24]. The tensile tests were performed in quasi-static loading conditions (2 mm/min) at room temperature, using a 100 kN universal testing machine Instron 4206. The local strain fields of the specimens tested, which were loaded transversely to the welding direction (skin direction), were acquired by an optical strain gauge GOM Aramis 5M system with digital image correlation (DIC). The procedures to prepare the specimens and to process/analyse the strain data were detailed in Leitão et al. [25].

**Figure 3:**
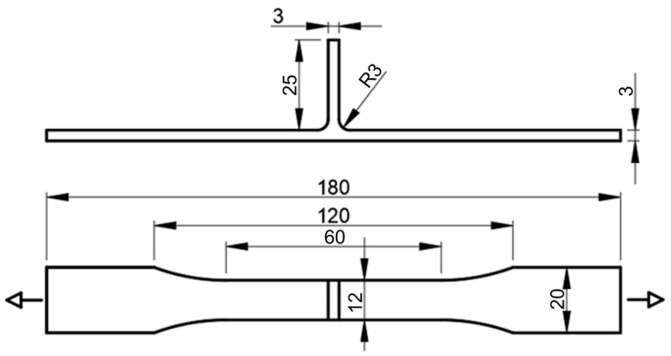
Fatigue specimen and loading conditions.

Fatigue specimens were removed transversely to the welding direction, with dimensions of 180 × 20 mm (Figure 3). The specimens were machined in a dog-bone shape in order to encourage failure in the weld region. The flash on the weld surface was removed and the edges were rounded and polished to avoid a concentration of surface stress and initiation of the crack. The load was applied in the skin direction transversely to the welding direction, and two fatigue specimens were tested for each load level applied. The fatigue tests were carried out using an Instron servo-hydraulic machine coupled to an Instron Fast Track 8800 acquisition and control system. The stress range varied between 150 to 200 MPa with a frequency of 15–25 Hz according to the load level applied, and the stress ratio was set to 0.02. The fracture surface of the fatigue specimens was analysed by scanning electron microscopy (SEM) using a Zeiss MERLIN field emission scanning electron microscope.

## 3. Results and Discussion

### 3.1. Welding Torque and Temperature

**Figure 4:**
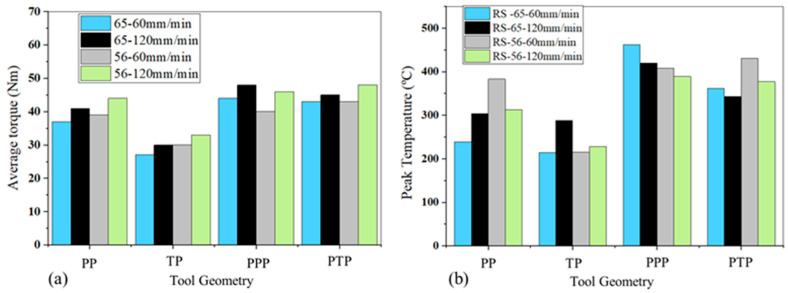
Process outputs for dissimilar welding: (**a**) average torque; (**b**) peak temperature.

**Figure 5:**
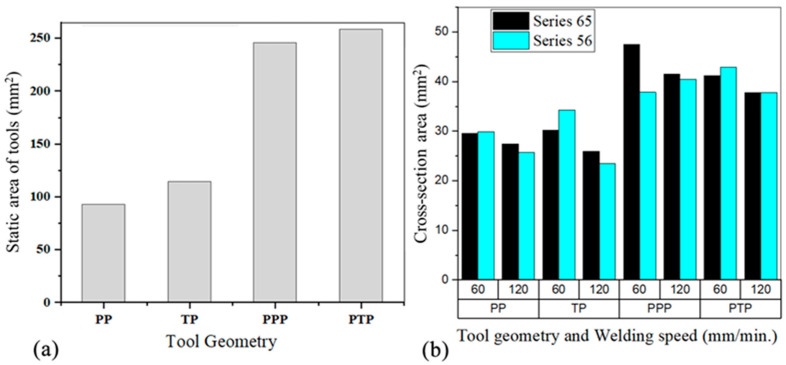
(**a**) Friction area of each tool pin; (**b**) cross section area of the stir zone according to the tool geometry and welding speed.

Figure 4a,b show the average torque and the peak temperature values obtained during dissimilar welding. The average torque was computed considering only the steady-state torque evolution, while the temperature values are peak temperature measurements performed on the retreating side of the welds. It was found that although the temperatures reached on the advancing side were higher, the thermocouples placed on the retreating side were less affected by the passage of the tool. It can be observed in Figure 4a that the highest torque values were registered in the welds produced with the progressive pin tools. However, Figure 4b shows that the highest peak temperature values were also registered in these welds, which does not agree with the conventional torque-temperature correlation, that is, when the temperature increases the torque required to deform the material decreases [10]. The temperature reached in the weld is governed by the heat input in the process, which depends on the welding parameters, such as the rotation and welding speeds and axial force of the tool, but also on its geometry and sticking/sliding conditions [26], specifically, the area of friction between the tool and the material being welded. In the current study, as the diameter of the shoulder of all the tools was approximately the same, the difference in the friction area between the tools came from the pin area. Figure 5a represents the friction area of each tool pin, already shown in Table 3, while Figure 5b shows the cross-section area of the stir zone for dissimilar welds.

The higher friction area between the progressive pin tools and the material to weld (Figure 5a) promoted an increase of the heat-input during welding, which reduced the flow stress of the material, as Siddiqui et al. [27] mentioned. Although the lower flow stress was expected to decrease the torque, progressive pin tools provided an increase in the amount of material dragged by the tool at each revolution, as the tools’ pin volumes suggest (Table 3) and the weld’s cross sections in Figure 5b show, and therefore the torque increased. There was no significant difference in terms of torque and even peak temperatures between the progressive pin tools (PPP and PTP) (Figure 4) because their friction areas are very similar and led to welds with similar cross-sections (Figure 5).

It can also be seen in Figure 4a that the average torque in dissimilar welding depends on the position of the base material, especially for single-pin tools (PP and TP). Higher torque values tended to be registered when AA5083 was welded as the skin, although the justification was less clear when looking at the cross section of the stir zone (Figure 5b). The base materials have quite different mechanical behaviour under high temperature and strain rate conditions. While AA6082 experiences strong softening under high temperature and strain rate conditions, AA5083 presents steady flow behaviour at high temperatures, but it is sensitive to moderate hardening at high strain rates [20,28]. When conventional tools are used, the shoulder-driven volume is much larger than the pin-driven volume. As the shoulder mostly contacts with the skin plate, higher flow stresses are experienced during the welding of the 56 series, which increases the torque. As the differences in the shoulder and pin-driven volumes decrease in dissimilar welding with the progressive pin tools, the effect of the position of the base material on the torque is less intense.

The average torque in dissimilar welding also increases with the traverse speed (Figure 4a). Similar findings were also reported by Banik et al. [29] and Aldanondo et al. [30], although Arora et al. [9] stated that torque is little influenced by welding speed. In most of the cases, the evolution of the temperature with the traverse speed supports the variation in torque, that is, the peak temperature decreases with the increase of welding speed (Figure 4b), and the base material presents higher material flow stresses [8], thus requiring greater torque. However, Figure 4b also shows that, in the case of the 65-weld series performed with the pyramidal pin tool (PP) and the tapered threaded pin tool (TP), an increase in the peak temperature occurred with the increase in the welding speed from 60 mm/min to 120 mm/min. This happened in the first case because there was an increase in tool penetration depth of 0.1 mm, and in the second by 0.5 mm, when the welding speed was changed from 60 mm/min to 120 mm/min. This clearly shows the influence of the tool penetration depth on the temperature developed in the weld. 

**Figure 6:**
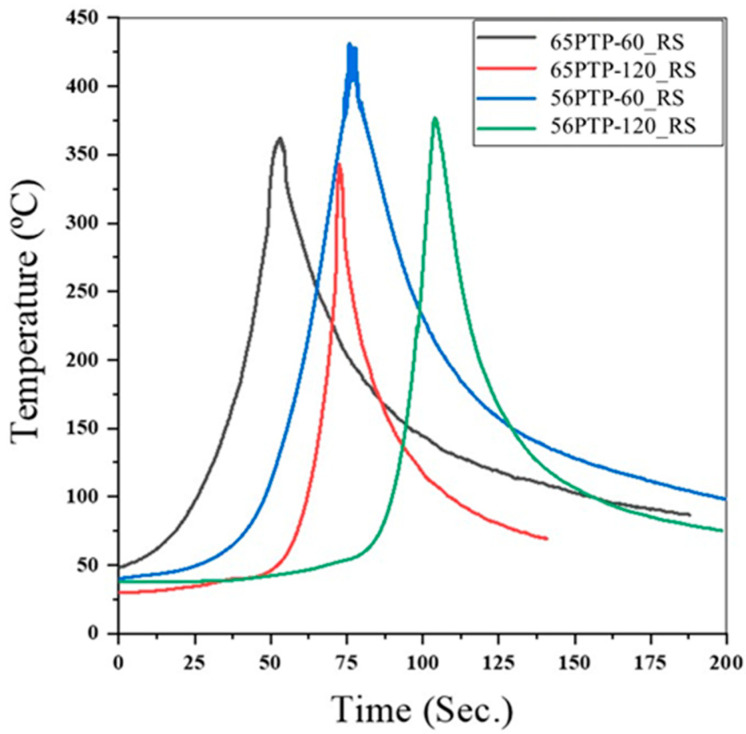
Thermal cycles measured on the retreating side of welds produced with different welding speeds and combination of materials.

Figure 6 illustrates the temperature variation with welding speed, measured on the retreating side, for welds performed with the progressive conical threaded tool (PTP) better. This figure also shows that in combination 56, higher peak temperatures were reached than in 65, although the recorded torque values were also higher (Figure 4a). This shows that the evolution of the torque cannot be interpreted exclusively based on the heat-input conditions; the tool/material contact conditions (sticking/sliding) [9], volume of material around the pin, and thermomechanical behaviour of the materials must also be considered.

**Figure 7:**
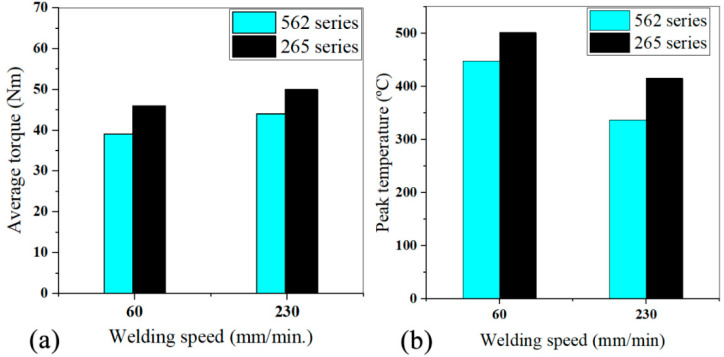
Process outputs for tri-dissimilar welding: (**a**) average torque; (**b**) peak temperature.

The tri-dissimilar welds were carried out with the progressive threaded pin tool (PTP), but varying the position of the skin plates (AA5083 and AA2017), either on the advancing side or on the retreating side, and with welding speeds of 60 mm/min and 230 mm/min. The average torque of the tri-dissimilar welds was also found to depend on the position of the base material, as the highest torque values were registered for the 265 welds (Figure 7a), that is, when the strongest material was placed on the advancing side. Figure 7b shows that the peak temperature difference was about 50 °C for the lowest welding speed, and this difference tended to increase for the highest speed. However, the conventional torque-temperature correlation was not observed in this case. The higher peak temperatures were not registered in the welds for which the lower torque values were measured (Figure 7), which confirms that torque is not exclusively governed by the heat-input during welding. Welding with AA2017 on the advancing side resulted in higher average torque values, even for welds produced under higher heat-input conditions.

**Figure 8:**
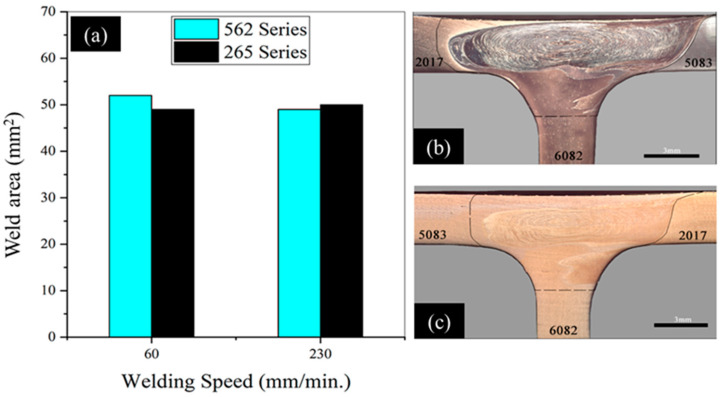
(**a**) Cross section areas of tri-dissimilar welds; (**b**) 562TP-60 (A = 52 mm^2^); (**c**) 265TP60 (A = 49 mm^2^).

Figure 7a also shows that the increase in welding speed increased torque, which is compatible with the reduction in peak temperature registered for any of the material combinations studied (Figure 7b). The increase in torque is also compatible with the increase in the specific volume of the material moved by the tool (V), this being given by the product of the cross section (A) by the welding speed (v) (V = Av). In fact, although the weld’s cross section (A) suffered a slight reduction with increasing speed (Figure 8), V greatly increased due to the large increase in v.

### 3.2. Weld Morphology

#### 3.2.1. Dissimilar Welds

**Figure 9:**
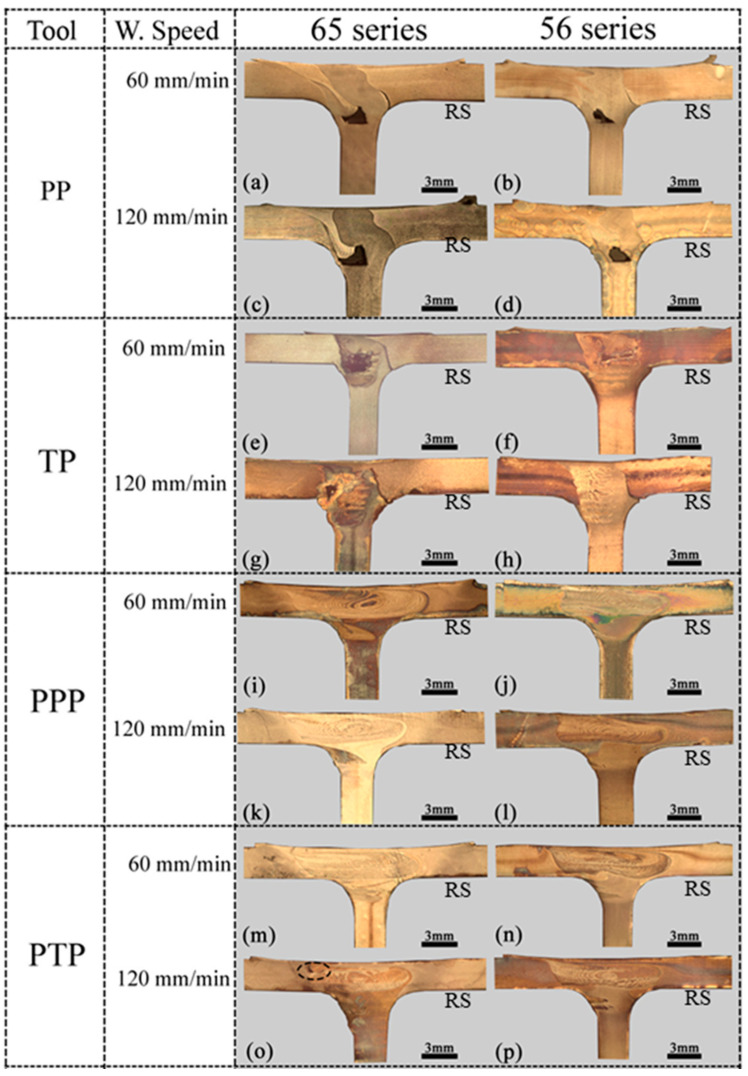
Cross-section macrographs of the dissimilar welds for different welding parameters.

Macrographs of the transverse cross-section of the dissimilar welds in the 65 and 56 series are shown in Figure 9, which illustrates the welds produced with varying pin profiles. Welds of Figure 9a–h were produced with threaded pin (TP) and pyramidal pin (PP) tools. It can be observed that the use of simple pin tools (TP and PP) promoted the formation of large void and/or lack of bonding (kissing bond) defects in the stir region of the dissimilar welds, regardless of the welding speed and the relative position of the base materials. Furthermore, the figures also show that larger defects were formed by a welding speed of 60 mm/min with a TP tool and when positioning the AA6082 alloy as the skin (Figure 9e). It was observed that the defect size tended to increase with a PP tool at the higher welding speed of 120 mm/min. By increasing the welding speed, the heat input per unit weld length decreased, more mm per tool rotation, which led to poor material plasticization and thermo-mechanical interaction between the tool and the material flow. For a threaded-pin tool, the inverse occurs. Sun and Wu [31] stated that threads improve the material flow close to the pin tip and broaden the thermomechanically affected zone. Figure 5b shows that the welds made with a threaded pin tool and a welding speed of 60 mm/min have a larger cross-sectional area than those made with a pyramidal pin, but this weld is the one with the largest cavity (Figure 9e). Discounting the cavity area substantially reduces the cross-sectional area of the weld, and hence the volume of material moved by the tool. On the other hand, pins with flat features produce a greater volume of material moved [32], which is what is observed in the welds performed with the pyramid tool (Figure 5b). When positioning the AA5083 alloy as the skin (56 series), a slight improvement in the dimensions of the defect was observed, but not enough to remove the defects. Furthermore, defects, either cavities or lack of bonding, occur in the fillet zone, which means that the volume of material moved by the tool in the zone is insufficient to prevent the formation of defects. The tool geometry influenced the location of the defects. Defects caused by the PP were located further away from the shoulder zone interaction. 

However, regardless of the specific design, it can be inferred that tools with a simple pin are not a viable solution for producing dissimilar T welds in AA5082 and AA6082, as these tools do not provide the intense mixing of the materials required to achieve non-defective joints. The cavity observed in dissimilar welds is related with a lower heat input, which results in insufficient plasticised material being deposited behind the tool [33].

On the other hand, the tools with a progressive pin (PPP and PTP), and regardless of the location of the base materials, allowed the production of welds without defects and with the formation of large onion-ring structures (Figure 9i–p). The exception is Figure 9o, which has a small void on the retreating side due to insufficient tool sinking. The defect free welds registered with progressive pin tools have two main reasons. First, the local softening of the materials makes the vertical material flow more efficient, promoting the formation of intercalated layers of the welded materials and abolishing the formation of defects. Figure 4b shows that the peak temperatures induced by these tools are higher than those of single-pin tools.

The second reason is related to differences in the volume of material moved by the tools. The pyramid progressive pin tool (PPP) has a static volume 3.4 times greater than the pyramid pin tool (PP) and provided about 1.4 times larger weld cross sections for welds 65–60. The progressive threaded pin tool (PTP) has 2.4 times the static pin volume of the tapered threaded pin (TP) and provided about 1.4 times the weld sections for the same type of welds.

Therefore, the progressive pin tools promote much greater dragging of material around the pin during the welding process, increasing the torque and heat input and reducing the likelihood of the occurrence of defects such as voids and/or lack of bonding.

#### 3.2.2. Tri-Dissimilar Welds

**Figure 10:**
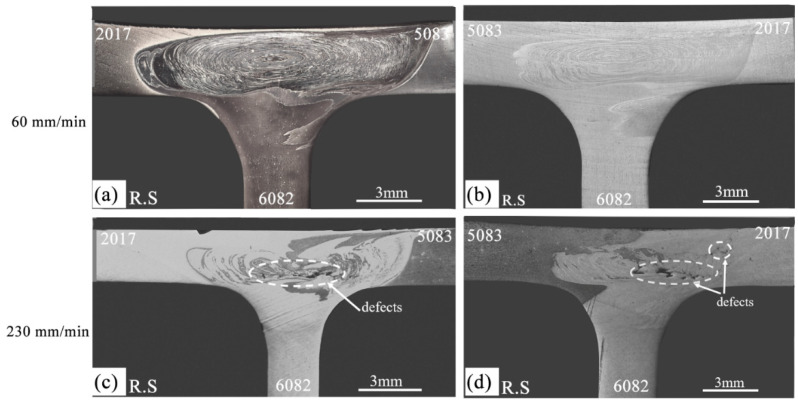
Cross section macrographs of the tri-dissimilar welds: (**a**) 562TP-60-6.8; (**b**) 265TP-60-7; (**c**) 562TP-230-7; (**d**) 265TP-230-7.

From the macrographs of tri-dissimilar welds, which are shown in Figure 10, it can be observed that sound macrostructures were achieved by welding with a traverse speed of 60 mm/min. In fact, regardless of the position of the base material, the cross-sections were defect-free and large onion-ring structures were formed in the weld nugget, as illustrated in Figure 10a,b. However, the figures also show that the increase in the traverse speed to 230 mm/min promoted the formation of defective structures in the nugget and removed the onion-ring structures (Figure 10c,d), which were replaced by chaotic mixing patterns. The detrimental effect of high traverse speed values on the morphology of welds produced by FSW has already been discussed by Kadian and Biswas [34] for butt joints and by Manuel et al. [22] for T-joints.

Figure 10c,d shows that the formation of these defects occurs at the boundary between the skin and the stinger. This is precisely the zone of interaction between the material flow induced by the cylindrical threaded part of the pin and the flow originated by the conical threaded part of the pin, as shown by Manuel et al. [21]. The formation of these defects is due to the insufficient interaction between these fluxes, caused by less plasticization of the materials, because the temperatures reached in the zone decrease with the increase in the welding speed, as shown in Figure 7b. Furthermore, the reduced space between the tool and the dies [21] constrains the upward flow of material induced by the conical threaded part of the pin.

### 3.3. Microstuctures in the Stir Zone

**Figure 11:**
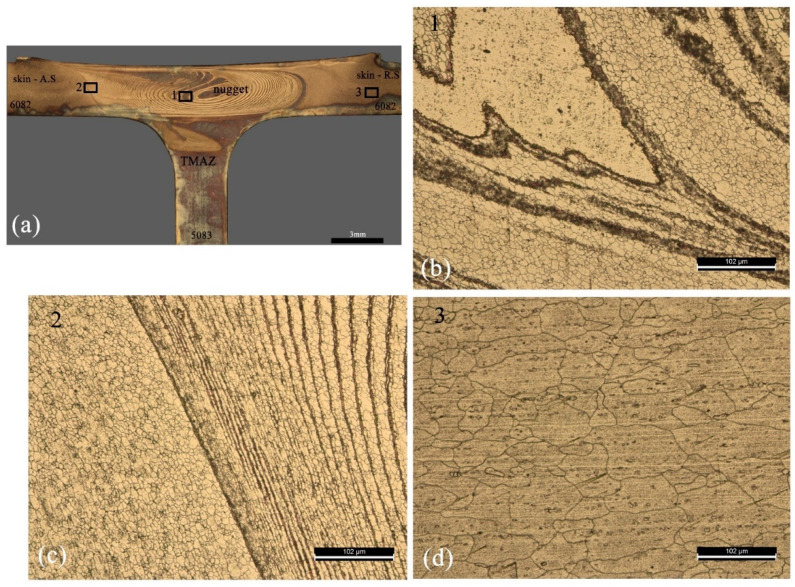
Stir zone of the 65PP-60 dissimilar welding: (**a**) weld cross section; (**b**–**d**) microstructure in Zones 1, 2, and 3.

The microstructure of the stir zone results from complex thermomechanical conditions, with the materials involved subjected to temperatures, degrees of deformation, and deformation rates that vary with the location in the nugget. Figure 11 illustrates some details of the stir zone of a 65PP-60 dissimilar weld. Figure 11a shows a macrograph of the weld and the location of the details illustrated in the following figures. Figure 11b shows a detail of zone 1 of the nugget, which illustrates the complexity of the flow of the two materials in the zone. The observation of these microstructures is difficult due to this mixing complexity, but mainly because the etchants are not effective simultaneously for both materials. The areas where grain is visible correspond to the AA6082 alloy, while the non-etched areas to AA5083. The figure shows that there was a great grain refinement in this zone when compared with the base material (AA6082), illustrated in Figure 11d. The grain in the nugget had an average size of 7.7 + 0.6 µm, while the base material did not have a uniform grain with an average size in the rolling direction of 56.7 + 31.7 µm. The grain was, however, not uniform in the nugget, as illustrated in Figure 11c. This figure shows that the grain decreased from about 8.2 µm to 3.2 µm when comparing the right side of the image with the central area, where very close deformation lines appeared. In this zone, the grains were very fine and elongated according to the direction of the deformation. In the area to the left of the image, where there seems to have been a lower degree of deformation, the recrystallisation [35] provided a more rounded and larger grain, of about 7.8 ± 2.9 µm. In the case of dissimilar welds, it is therefore difficult to characterise the grain size in the stir zone due to the variability that is observed. Changing the tool geometry did not significantly alter the microstructure in the nugget. Increasing the welding speed further refined the grain to values down to 5.7 µm.

**Figure 12:**
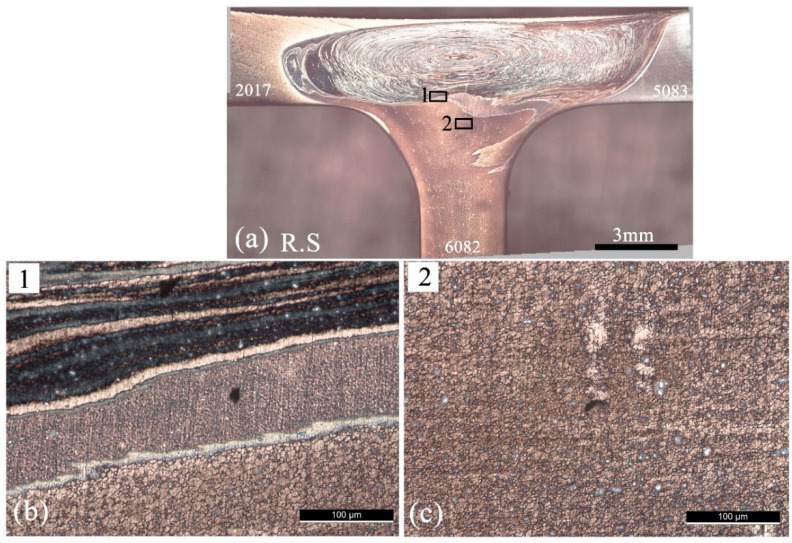
Stir zone microstructures of the 562TP-60 tri-dissimilar welding: (**a**) weld cross section; (**b**) and (**c**) microstructure in Zones 1 and 2.

Figure 12 illustrates some microstructural details of a weld of the three alloys considered, specifically 562TP-60. Figure 12a gives the location of the details illustrated in Figure 12b,c. Figure 12b corresponds to an onion ring zone, where the fluxes of the three materials are illustrated. The upper dark bands correspond to AA2017, which is over-etched, the middle band to AA5083, which is not properly etched, and the lower band to AA6082 that is properly etched. The white zones are zones of interaction between the two alloys, which have not been etched. In tri-dissimilar welds, etching is even more difficult, as conventional reagents behave differently from those used for homogeneous welds. Figure 12c illustrates the microstructure of a part of the nugget, where the formation of a very refined grain, of about 6 ± 2.7 µm, occurred. The higher temperatures reached in these welds (Figure 7b) due to the use of a progressive pin tool suggested the formation of coarser grain. The plastic deformation rate must have conditioned this growth, as mentioned above. The increase in welding speed allowed the refinement of the grain in the nugget to values of about 4 µm.

### 3.4. Weld Mechanical Behaviour

#### 3.4.1. Microhardness

Single-pin tools did not provide sound dissimilar welds, so these welds were not mechanically tested. Progressive pin tools delivered dissimilar welds free of defects, but whose effect on the mechanical behaviour, for the range of parameters tested, has been previously analysed [22]. Therefore, only the analysis of the mechanical behaviour of the tri-dissimilar welds is presented.

**Figure 13:**
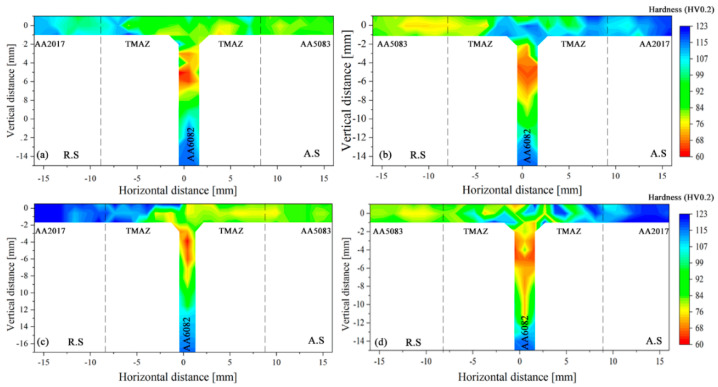
Skin and stringer hardness for the 562 and 265 welds: (**a**) 562TP-60; (**b**) 265TP-60; (**c**) 562TP-230; (**d**) 265TP-230.

Hardness maps of tri-dissimilar welds are presented in Figure 13, where the advancing and retreating sides are indicated by AS and RS, respectively. Comparing Figure 13a with Figure 13c, which corresponds to welds performed in series 562 with welding speeds of 60 mm/min and 230 mm/min, respectively, significant differences can be observed, mainly in the heat-affected zones on the sides of AA2017 and AA6082. Actually, the weld carried out with 60 mm/min presents a hardness reduction from 120 HV0.2 to about 80 HV0.2 on the AA2017 side, in an extension of nearly 13 mm, while for the welding carried out at 230 mm/min this reduction extended to about 5 mm. In the stringer (AA6082 side), the drop in hardness was greater, from about 115 to 60 HV0.2, but to a lesser extent, in the weld performed at a higher speed. These losses in hardness were expected and have already been analysed by other authors [36]. A dissolution of strengthening precipitation occurs when heat-treatable alloys are exposed at higher temperature, which leads to hardness drops in HAZ and TMAZ. The hardness reduction is more notable in AA6082 because it was in temper T6, although the location of this zone was further away from the shoulder of the tool. Most of the heat is generated by the tool shoulder [37]. In the stir zone, the hardness distribution is heterogeneous due to the combined effect of the dissolution of hardening precipitates and the mixture of the three alloys. The mix of the three alloys in the stir zone is very complex, as mentioned in Section 3.3 and illustrated in Figure 12. On the AA5083 side, there was a slight hardness variation because the base material had a soft temper.

In case of the series 265 presented in Figure 13b,d, a significant fluctuation of hardness values, between 71 and 117 HV0.2, was registered in the TMAZ and nugget zone for 60 and 230 mm/min welding speeds. This may be attributed to the complex flow of material that results in a heterogeneous mixture of the three materials in the stir zone as shown in the previous section. In the heat-affected zones the effects are like those recorded for the 562 series, although the loss of hardness areas in the stringer of the 265 series are more extensive. This is compatible with the higher peak temperatures recorded for this weld series (Figure 7b). 

In summary, it was found that the distribution of hardness in the weld is influenced by the properties of the base materials and their relative position in the joint. When AA2017 was on the advancing side, a more significant effect on hardness under the same welding conditions was registered.

#### 3.4.2. Ultimate Tensile Test

The ultimate tensile strength (UTS) of the tri-dissimilar welds is shown in Table 6. As three specimens were tested per weld, the UTS values displayed in the table correspond to the average of the results of all the specimens tested. The table also shows the fracture zone and the weld strength efficiency, which was computed through the ratio between the weld’s UTS and the UTS of the base material with the lowest strength (AA5083). It can be observed that most of the welds presented efficiency values over 93%, which indicates that the welding parameters allowed the production of welds with an improved tensile behaviour. Even so, flow defects were also formed in the nugget of the 562TP-230 weld, but they had no effect on its mechanical strength.

**Figure 14:**
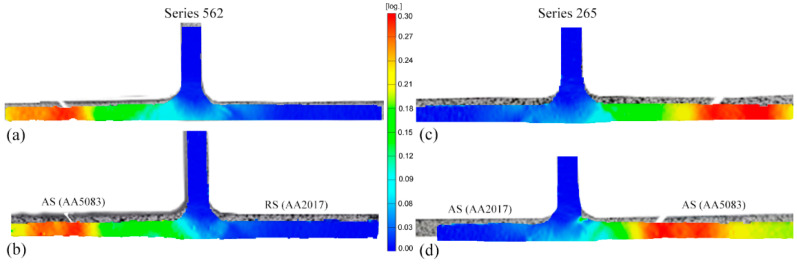
Strain distribution maps: (**a**) 562TP-60; (**b**) 265TP-60; (**c**) 562TP-230; (**d**) 265TP-230.

The strength efficiency of the 562TP-230 weld was slightly higher than the efficiency of the remaining ones. However, as illustrated in Figure 14, which shows the strain distribution maps obtained by digital image correlation at maximum load, all the specimens tested failed at the AA5083, regardless of the traverse speed or the position of the base material. Taking into consideration the efficiency values of the specimens, which are less than 100%, it can be stated that the welds failed in the heat-affected zone. The heat input during welding promoted the slight softening of AA5083, and therefore, the strain was found to concentrate in this alloy during testing.

#### 3.4.3. Fatigue Tests

**Figure 15:**
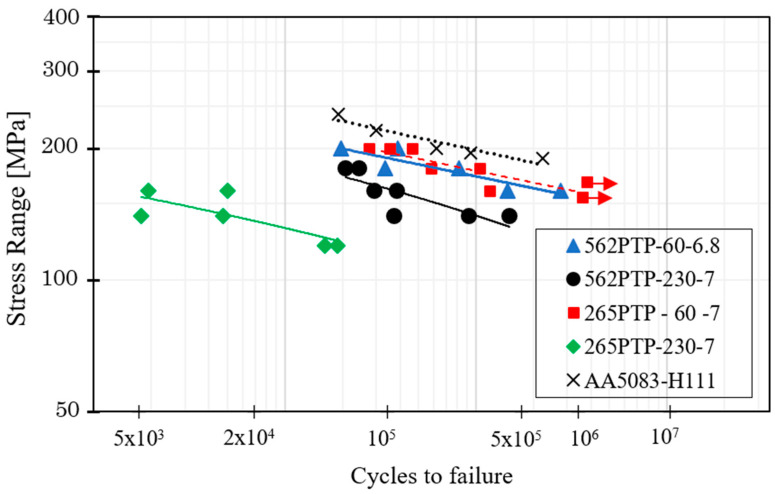
Fatigue S-N curves obtained for the tri-dissimilar welds and the AA5083 base material.

Figure 15 shows the results of the fatigue tests for the tri-dissimilar welds and AA5083 base material. Horizontal arrows in the graph represent that the fatigue specimens did not break after more than half a million cycles. The red curve is shown in dashed lines for this reason. From the figure, it can be observed that all the welds presented a lower fatigue strength than the base material.

Although FSW is a solid-state technology, in which the heat generation is lower than in the conventional welding processes, the material strength was affected by the welding thermal cycle and, consequently, the fatigue behaviour of the welds was conditioned, even for the non-defective joints. However, regardless of the position of the base material, the fatigue behaviour of the welds produced with 60 mm/min, which were free of defects (Figure 10a), was better than that of the welds produced with a higher traverse speed. The fatigue behaviour of the welds produced with a lower traverse speed, which were found to fracture in the HAZ (AA5083 side), only decreased 10% (265TP-60) and 13% (562TP-60) at 10^6^ cycles when compared with the base material (AA5083). Therefore, the 265 weld series has slightly better fatigue strength than the 562 series. On the other hand, the fatigue strength of the welds produced with a traverse speed of 230 mm/min decreased 27% (562TP-230) and 47% (256TP-230). In good agreement with this, the fracture of these welds occurred in the nugget, where flow defects were observed (Figure 10c,d). Specifically, the flow defects identified in the 265TP-230 weld were formed near the fillet region, which seriously conditioned the fatigue behaviour of this weld. In turn, the defects formed in the 562PTP-230 weld, despite not affecting the weld tensile behaviour, which was the best among the different welds, strongly conditioned the fatigue behaviour. In fact, the presence of defects in these welds is crucial [20,38].

Table 7 presents the equations that describe the S-N curves obtained from the series of welds illustrated in Figure 15. The slope “m” indicates the prevalence of the initiation of the crack and propagation until fracture. The fatigue strength values were determined at one million cycles. The results indicate that welds with higher value of the factor “m” present better fatigue resistance such the welds 562-60 and 265-60. The curves of the welds performed at the highest speed showed greater dispersion, see R^2^, due to the presence of defects, as mentioned below.

**Figure 16:**
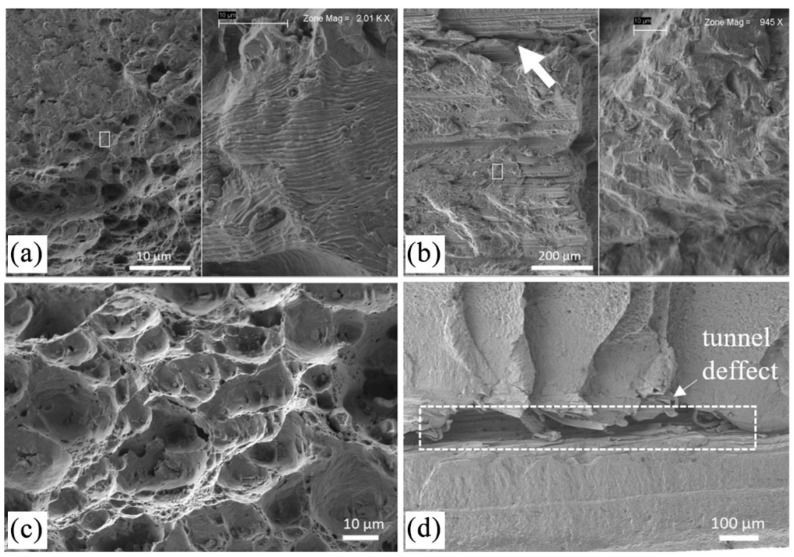
Fracture surfaces of the fatigue-tested specimens: (**a**) 562TP-60, (**b**) 562TP-230, (**c**) 265TP-60, (**d**) 265TP-230.

The results of the fractographic analyses performed on the fatigue specimens are shown in Figure 16. From the figure, it can be observed that the welds produced with a lower traverse speed presented a ductile fracture (Figure 16a,c), which is in good agreement with the better fatigue behaviour of these welds. Specifically, the detail highlighted on the fracture surface of the 562TP-60 weld (Figure 16a) allows us to observe the striation pattern associated with the crack growth. The high fatigue lives for the 562TP-60 welds of 802,067 cycles is clear evidence of the lack of defects in these welds.

Regarding the welds produced with a higher welding speed (Figure 16b,d), defects were observed on the fracture surfaces, which, as reported above, limited the fatigue behaviour of these joints. Although the stress range applied was low (120 and 140 MPa), the specimens fractured with a very low number of cycles of 54,421 and 265,159, respectively.

## 4. Conclusions

The current investigation analysed the influence of tool geometry, process parameters, and position of the materials on the temperature, torque, and quality of the weld of two and tri-dissimilar aluminium alloys. The following conclusions were drawn:

- Tools with a single pin, whether pyramidal or tapered threaded, generate lower peak temperature and torque than tools with a progressive pin. This is caused by the smaller friction area and smaller volume of material moved by the single pin tools;
- Placing the stronger alloy on the advancing side leads to higher welding temperature and torque, while increasing the welding speed reduces the peak temperature but increases the torque used;
- Single-pin tools do not provide sound welds, unlike progressive pin tools, and this is related to the difference in the temperature and volume of material dragged by the different tools;
- The nugget of the welds has a very refined, but not uniform, grain structure whose size is conditioned by the heat input, but also by the way in which the local plastic deformation of the materials occurs;
- The increase in welding speed causes the appearance of defects in tri-dissimilar welds, even when using a tool with a progressive pin, which causes a reduction in fatigue strength, especially when the more resistant alloy is placed on the advancing side.

## Figures and Tables

**Table 1:** Chemical composition of the base materials (wt.%).

Alloys	Cu	Mg	Mn	Fe	Si	Zn	Ti
AA2017-T4	4.5	0.8	1.0	0.7	0.8	0.25	0.15
AA5083-H111	0.025	4.5	0.57	0.18	0.09	0.01	0.01
AA6082-T6	0.09	0.6	1.0	0.44	0.81	0.08	0.03

**Table 2:** Mechanical properties of the base materials.

Properties	AA2017-T4	AA5083-H111	AA6082-T6
Ultimate tensile strength (MPa)	427.0	317.5	321.0
Tensile yield strength (MPa)	276.0	158.0	288.0
Elongation at failure (%)	22.0	10.4	8.6
Vickers hardness (HV 0.2)	118.0	83.5	116.0

**Table 3:** Volume and friction area of tool pins (probes).

**Tool pin** **geometry**	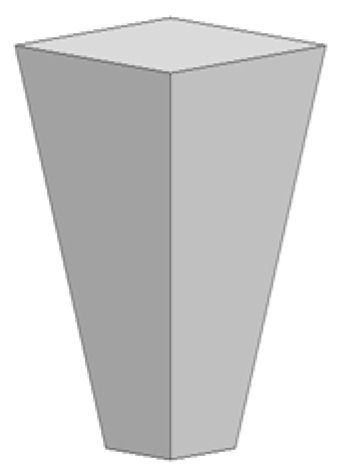	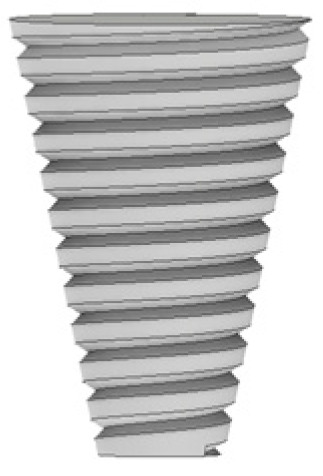	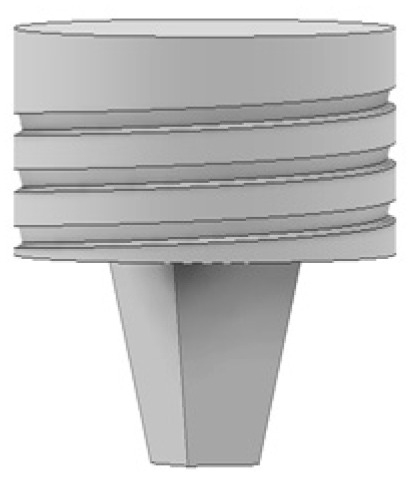	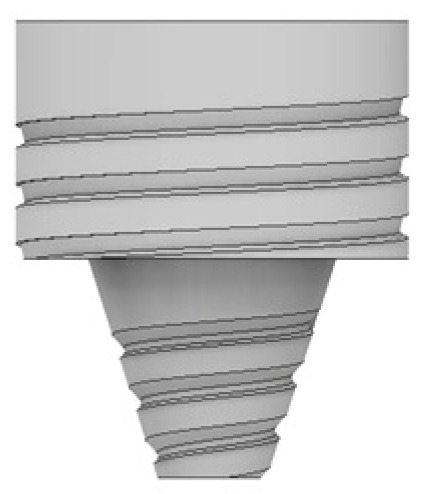
**Volume (mm^3^)**	55.33	86.08	188.97	198.8
**Friction area (mm^2^)**	92.4	114.12	245.36	258.13

**Table 4:** Welding parameters for dissimilar welds.

Welds	Tool	Rotation Speed (rpm)	Traverse Speed (mm/min)
65P-60	PP	500	60
65P-120			120
56P-60			60
56P-120			120
65T-60	TP	500	60
65T-120			120
56T-60			60
56T-120			120
65PP-60	PPP	500	60
65PP-120			120
56PP-60			60
56PP-120			120
65TP-60	PTP	500	60
65TP-120			120
56TP-60			60
56TP-120			120

**Table 5:** Welding parameters for tri-dissimilar welds.

Welds	Tool	Rotation Speed (rpm)	Traverse Speed (mm/min)
562TP-60	PTP	500	60
562TP-230			230
265TP-60	PTP	500	60
265TP-230			230

**Table 6:** Tensile properties of the welds.

Weld	UTS [MPa]	Strength Efficiency [%]	Zone of Fracture
562TP-60	295.7	93.7	HAZ (AA5083)
562TP-230	304.4	96.1	HAZ (AA5083)
265TP-60	295.6	93.2	HAZ (AA5083)
265TP-230	263.2	82.8	HAZ (AA5083)

**Table 7:** Equations of S-N curves for fatigue strength at 10^6^ cycles.

Series	Line Equation	Fatigue Strength at 10^6^ Cycles (MPa)	Slope “m”	R^2^
562TP-60	σ = 537.98 N^−0.09^	155.16	11.11	0.79
562TP-230	σ = 692.67 N^−0.127^	119.82	7.87	0.66
265TP-60	σ = 554.29 N^−0.09^	159.86	11.11	0.78
265TP-230	σ = 365.6 N^−0.1^	91.39	9.90	0.55
AA5083	σ = 653.15 N^−0.095^	175.80	10.53	0.89

## Data Availability

The data presented in this study are available on reasonable request from the corresponding author.
